# Generation and Analysis of Expressed Sequence Tags (ESTs) from Halophyte *Atriplex canescens* to Explore Salt-Responsive Related Genes

**DOI:** 10.3390/ijms150611172

**Published:** 2014-06-23

**Authors:** Jingtao Li, Xinhua Sun, Gang Yu, Chengguo Jia, Jinliang Liu, Hongyu Pan

**Affiliations:** College of Plant Science, Jilin University, Changchun 130062, China; E-Mails: lijingtao789@126.com (J.Li); xinhua_sun@126.com (X.S.); chrisyu_gang@126.com (G.Y.); jiacg@jlu.edu.cn (C.J.); jlliu@jlu.edu.cn (J.Liu)

**Keywords:** *Atriplex canescens*, cDNA library, expressed sequence tags, salt stress tolerance, SSRs

## Abstract

Little information is available on gene expression profiling of halophyte *A. canescens*. To elucidate the molecular mechanism for stress tolerance in *A. canescens*, a full-length complementary DNA library was generated from *A. canescens* exposed to 400 mM NaCl, and provided 343 high-quality ESTs. In an evaluation of 343 valid EST sequences in the cDNA library, 197 unigenes were assembled, among which 190 unigenes (83.1% ESTs) were identified according to their significant similarities with proteins of known functions. All the 343 EST sequences have been deposited in the dbEST GenBank under accession numbers JZ535802 to JZ536144. According to *Arabidopsis* MIPS functional category and GO classifications, we identified 193 unigenes of the 311 annotations EST, representing 72 non-redundant unigenes sharing similarities with genes related to the defense response. The sets of ESTs obtained provide a rich genetic resource and 17 up-regulated genes related to salt stress resistance were identified by qRT-PCR. Six of these genes may contribute crucially to earlier and later stage salt stress resistance. Additionally, among the 343 unigenes sequences, 22 simple sequence repeats (SSRs) were also identified contributing to the study of *A. canescens* resources.

## 1. Introduction

Salinity is a soil condition characterized by a high concentration of soluble salts. Soil salinity stresses plants in two ways: a rapid, osmotic phase that inhibits growth of young leaves, and a slower, ionic phase that accelerates senescence of mature leaves. All plants have evolved mechanisms to regulate salt accumulation and to select against it in favor of other nutrients commonly present in low concentrations, and to tolerate the low soil water potential caused by salinity, as well as by drought. Plant adaptations to salinity are of three distinct types: osmotic stress tolerance; Na^+^ exclusion; and tissue tolerance [1]. Agricultural losses caused by salinity in many arid and semiarid regions are difficult to assess, but estimated to be substantial and expected to increase with time. Therefore, cultivation of salt-tolerant crops, or halophytes, on saline soil has significant social and economic potential that needs to be further explored and developed [2]. There have been many physiological and biochemical studies performed to investigate salt tolerance in plants; additionally some candidate genes have been transferred from one species of plants to another, and exhibited enhanced tolerance to various stimuli [3,4]. Since tolerance to salt stress is controlled by numerous genes, detailed insight into the processes requires an identification of novel genes, which can be facilitated by genomic approaches [5,6]

Salt stress and salt shock are two distinct phenomena, both triggered by the application of salt. If researchers want to study and create novel salt-tolerant plants, salt stress rather than salt shock is to be studied in preference. However, because the timescale of the treatment should extend from minutes (e.g., 30 min) to days (e.g., 1 week), for identification of genes with short- and longer-term responses, many researchers are still using salt shock in experiments. But it must be taken into consideration that total gene expression profiles, and expression changes in particular genes of interest, are very likely to be different following treatment with the same NaCl concentration applied to induce either salt stress or salt shock [7].

Genomic strategies such as expressed sequence tags (ESTs) approaches have been proved to be efficient, comparatively cheap, rapid and powerful means to identify novel genes (and proteins) regulated by environmental changes or stresses, especially in organisms whose reference genomic information is not available [8,9]. Large-scale cDNA sequencing projects and EST analysis have been conducted in a number of halophytes, such as *Puccinellia tenuiflora* [6], *Selaginella lepidophylla* [8], *Suaedu salsa* [10], *Thellungiella halophile* [11], *Mesembryanthemum crystallinum* [12], *Avicennia marina* [13] and *Limonium bicolor* [14], and their EST databases have been established to discover stress resistance genes and to determine the expression patterns of abiotic stress in different plants.

The halophytes *Atriplex* genus, members of the Chenopodiaceae, have been extensively used in physiological and molecular biological investigation to explore stress-related novel genes. Tolerance to salinity, drought, heavy metals and temperature are important characteristics for *Atriplex* species, especially tolerance to drought and salt [2]. And it is of great significance for generating tolerant crops via selective breeding or genetic engineering to elucidate its tolerance mechanisms. Currently, several stress-related genes have been isolated and characterized from *Atriplex* species to some extent. Transgenic rice overexpressing the *AgNHX1* gene from *A. gmelili* could survive after a short period of exposure to high concentrations of NaCl [15]. A choline monooxygenase gene isolated from *A. hortensis* ( *AhCMO*) has been used for glycinebetaine production in tobacco [16] and cotton plants [17] to improve their abiotic stress tolerance. A betaine aldehyde dehydrogenase gene from *A. hortensis* ( *AhBADH*) was introduced into tomato and trifoliate orange [18,19] and significantly improved salt tolerance in transgenic plants during growth. In transgenic tobacco, *AhDREB1* isolated from *A. hortensis* led to accumulation of its putative downstream genes and exhibited increasing stress tolerance [20].

The four-wing saltbush, *A. canescens*, has been extensively recommended as an excellent phytoremediation plants in saline-alkali and heavy-metal contaminated land [2]. This kind of saltbush could exhibit tolerance to salinity, drought, heavy metals and low temperature, which make *A. canescens* a source for exploring exclusive genes or new genetic mechanisms that could be applied for genetic manipulation of crops. However, little information is yet available on the global gene expression patterns of halophyte *A. canescens* [2]. Therefore, analysis of ESTs from *A. canescens* under saline stress is essential for elucidating the molecular mechanism of salt tolerance in *A. canescens*, which will provide useful information for the breeding and genetic engineering of salt tolerant crops as well.

Molecular markers play an important role in many aspects of plant breeding, such as identification of the genes responsible for desirable traits. Molecular markers have been widely used to map important genes and assist with the breeding of oil crops [21]. Compared with other types of molecular markers, SSRs have many advantages, such as simplicity, effectiveness, abundance, hypervariability, reproducibility, codominant inheritance, and extensive genomic coverage [22]. Based on the original sequences used to identify simple repeats, SSRs can be divided into genomic SSRs and EST-SSRs. Genomic SSRs are costly, labor-intensive, and time-consuming and the interspecific transferability of genomic SSRs is limited because of either a disappearance of the repeat region or degeneration of the primer binding sites [23]. Alternatively, EST-SSRs are derived from expressed sequences, which are more evolutionary conserved than noncoding sequences; therefore, EST-SSR markers have a relatively high transferability. In *A. canescens*, there are no EST-SSRs developed owing to lack of ESTs in public databases. Thus, a rapid and cost-effective approach to develop molecular markers for *A. canescens* is required and valuable according to its ESTs.

Here we report a preliminary study of ESTs analysis of *A. canescens* response to salt stress by constructing a full-length cDNA library. According to previous reports, ESTs sequencing has been established as a good platform to explore and study abiotic stress-related genes in plants [14]. In this study, a full-length cDNA library of *A. canescens*, exposed to 400 mM NaCl for 48 h, was constructed, and generated 343 high-quality ESTs, ultimately providing a view of transcript expression in *A. canescens* during salt induced stress. The sets of ESTs obtained provide a useful resource for identifying putative novel genes related to abiotic stress tolerance and as a reference for comparative genomics. Further, 23 potential genes related to salt stress tolerance were identified by qRT-PCR.

## 2. Results and Discussion

### 2.1. General Characteristics of the cDNA Library

**Figure 1:**
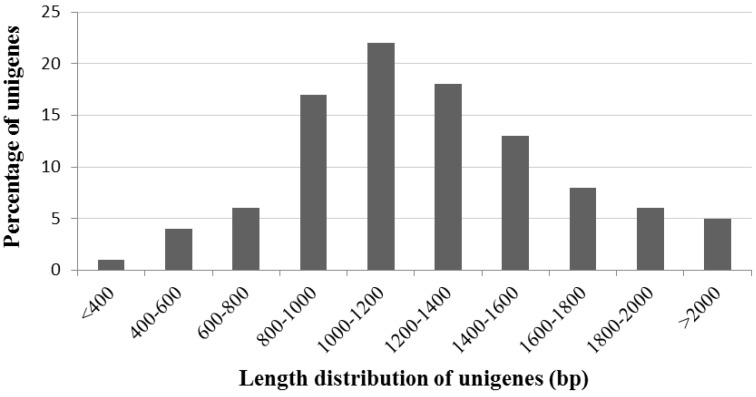
Comparison of unigene length.

In an effort to generate sequence data for *A. canescens*, a cDNA library was generated based on high quality RNA samples with three biological replicates (Figure S1A). The primary titer of the amplified library was 1.76 × 10^6^ CFU (Colony-Forming Units), with a recombinant rate of 91% for the original library, and the sizes of inserts ranged from 0.4 to 2.4 kb (Figure S1B). Then PCR was performed on approximately 500 colonies to investigate the average insert size, and the most abundant insert size is between 1000 and 1200 bp (Figure 1); and longer inserts were more likely to have BLASTX homologs in protein databases; 89.00% of the inserts over 800 bp had BLASTX homologs, while only 1% of the unigenes were shorter than 400 bp had homologs. These results indicate that our cDNA library was also qualified [24].

### 2.2. General Characteristics of A. canescens ESTs

**Figure 2:**
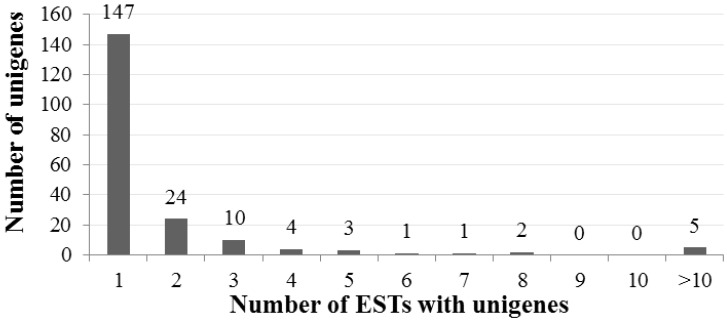
Distribution of ESTs with number of unigenes in *A. canescens*.

Approximately 500 clones were selected and randomly sequenced (BGI Corporation, Beijing, China); 413 clones were sequenced successfully to generate ESTs. Trimming of the short sequences (<100 bp), vector sequences, and poor-quality sequences resulted in 343 high-quality ESTs, constituting a total of 322,524 bases in the *A. canescens* sequence, with the G + C content 44.66%. The average read length of these ESTs was 940 bp (Table S1). The clustering of ESTs generated 50 contigs (containing 2 or more ESTs) and 147 singletons (containing only 1 EST), yielding 197 unigenes (Table S1). The redundancy of the library was calculated as 42.6% ((1 − Number of Unigenes/Number of ESTs) × 100%) [24]. The distribution of ESTs in unigenes after clustering was also generated (Figure 2). Fifty contigs had at least 2 ESTs, and among these, the largest group contains 24 ESTs (Figure 2). The results indicated that the library should be sufficient to meet the requirements for an EST analysis. All of the 343 high-quality ESTs have been deposited in the GenBank dbEST database under accession numbers JZ535802 to JZ536144.

### 2.3. Functional Annotation and Classification of A. Canescens Unigenes

**Figure 3:**
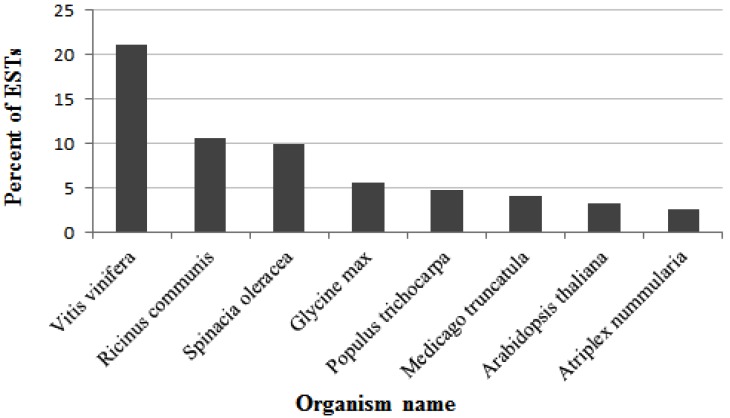
The seven most frequently matched plants according to the BLASTX EST search results. Percentages are with respect to the total set of non-redundant 343 transcripts.

The BLASTX search revealed that there were 343 ESTs (representing 197 unique genes) out of a total of 413 sequenced clones showing significant similarity (E-value < 10^−4^) to proteins in NCBI nr database. One hundred and ninety (285 ESTs) among the 197 unigenes (343 ESTs) were identified as known functions (83% ESTs). Unsurprisingly, the BLASTX search results also showed a bias towards plants sequences found in dicotyledons. The top 8 matched plants, starting from highest score were: *Vitis vinifera*, *Ricinus communis*, *Spinacia oleracea*, *Glycine max*, *Populus trichocarpa*, *Medicago truncatula*, *A. thaliana*, *A. nummularia* (Figure 3).

**Figure 4:**
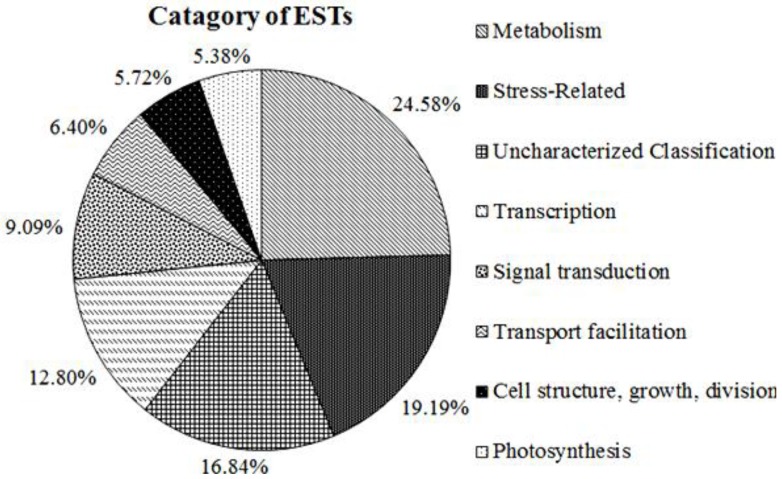
Ten functional categories among the 343 identified ESTs. All ESTs were assigned to functional category based not only on highest scoring BLASTX results but also on its covering extension according to the *Arabidopsis* MIPS functional category denomination. Percentages are with respect to the total set of ESTs with a high homology to known proteins.

The 343 ESTs that match sequences in the NCBI nr database were further divided into 8 functional categories based on the *Arabidopsis* MIPS functional category denomination (Figure 4). Those homologous to unnamed protein, uncharacterized protein, no significant similarity found, predicted protein and hypothetical proteins, were collectively designated “Uncharacterized Classification” (16.84%), which is consistent with the known functions content (83% ESTs) from the NCBI database. The other 7 functional categories were metabolism (24.58%), Stress related (19.19%), Transcription (12.80%), Signal transduction (12.80%), Transport facilitation (9.09%), Cell structure, growth, division (6.40%), and Photosynthesis (5.72%).

In addition to providing a quick method for gene discovery, EST analysis is also a powerful tool for determining gene expression levels. By EST analysis, the genes abundantly expressed were identified. We found 10 most abundant genes (the copy number > 4) in the EST collection, which accounted for 32.28% of the total ESTs (Table S2). As expected, the most abundant sequences, Chlorophyll A/B binding protein related genes accounted for 2.62% of total ESTs, suggesting photosynthesis was still active in leaves of *A. canescens* under NaCl stress condition. Accordingly, glyceraldehyde 3-phosphate dehydrogenase, which represents 2.33% of the transcripts in *A*. *canescens*, was one of the most abundant transcripts of the transcriptome of poplar, Arabidopsis and maize, and is usually considered as a house-keeping gene [25,26,27]. Heat-shock proteins were also highly expressed in response to the abiotic stress. In addition, other sequences, such as that encoding eukaryotic elongation factor, show similarity to genes encoding proteins that assist in the elongation of protein during translation [28]. Interestingly, the frequency of genes matched to osmo-protectant synthesizing proteins, was lower than that of other functional groups (Table S2). One explanation for this may be that some of those genes were expressed early in response to the NaCl treatment and were no longer expressed at 48 h when transcripts were isolated [6].

### 2.4. Gene GO Classifications and Genes Potentially Involved in Abiotic Tolerance

**Figure 5:**
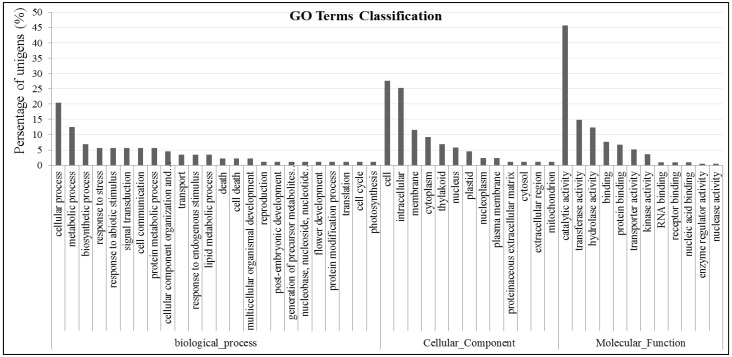
GO classification of the ESTs based on their biological functions, cellular components and molecular functions in the *A. canescens* cDNA library.

Sequences from the cDNA library was translated using BLASTX. The translated sequences were submitted to the Gene Ontology (GO) to identify signatures representing specific protein families or domains, and the corresponding GO terms ID [29]. The GO terms were further used to classify the gene products in functional GO categories and simplified into plant-specific annotations (GO classification) to obtain additional insights into the putative functions of unigenes. Of the 343 *A*. *canescens* ESTs, 311 were assigned GO terms in any category (biological, cellular, and molecular), and the other 32 ESTs (represent 4 unigenes) were uncharacterized proteins without GO terms annotations (Figure 5). We identified 193 unigenes from the 311 annotated ESTs, representing 72 non-redundant unigenes, that share similarities with genes related to defense and stress response according to GO classifications and previously published data [24]. These genes are involved in a variety of functional areas, such as response to stress and abiotic stimulus, cellular component organization and biogenesis, transport, response to endogenous stimulus, lipid metabolic process, cell death, thylakoid, protein binding, catalytic activity, transporter activity. The analyses of these genes may be important for revealing the saline tolerance mechanism of *A*. *canescens* (Table 1).

Expectedly, the ESTs involved in “response to stress and abiotic stimulus” in biological process ontology were highly abundant in the library, because the *A*. *canescens* genes were isolated from halophytes plant treated with salt stress, thereby confirming previous reports (Figure 5). Exposure to saline stress may result in the accumulation of low-molecular mass compounds in the cytosol, and it also stabilizes both the PSII complex and RuBisCo during photosynthesis under stress conditions [3]. Sugar transport protein and the sodium-bile acid cotransporter from the “transporter activity” section in molecular function ontology play similar roles in maintaining stable osmotic pressure [14]. The “Thylakoid” and “enzyme regulator activity” ESTs are also abundant, suggesting photosynthesis is still active under NaCl treatment.

Meanwhile, both glycophytes and halophytes cannot tolerate large amounts of salt in the cytoplasm. The greater salt tolerance in *Atriplex* species is related to the efficient transport and compartmentalisation of toxic Na^+^ ions to vacuole in shoots, which prevents the ionic damage of the cytoplasm. Therefore, the “signal transduction and cell communication”, “cellular component organization and biogenesis”, “transport”, “lipid metabolic process”, and “cell death” has been characterized and identified, and the related genes may play important roles in the stable osmotic pressure and response to the abiotic stress, such as stress-induced protein, non-specific lipid-transfer protein, abscisic acid stress ripening protein, leucine-rich repeat receptor-like protein kinase and so on. Transcription factor genes play important roles in stress survival by serving as master regulators of sets of downstream stress-responsive genes via binding to specific elements ( *cis*-elements) in target genes [2]. The ethylene response factor (ERF) transcription factor can be found from “response to endogenous stimulus” in biological process ontology.

In addition, several unigenes responsive to salt, cold and drought stresses were found in the library according to plant GO terms; these were classified as “protein binding”, “catalytic activity”, “kinase activity” and “receptor binding”. Heat-shock protein, glyceraldehyde-3-phosphate dehydrogenase, *S*-adenosylmethionine synthase, eukaryotic elongation factor, transforming growth factor and others were response to abiotic stresses [6,9,24].

**Table 1:** Genes potentially involved in salt tolerance in *A. canescens.*

Gene Accession NO.	Gene Description	Matching Organism	E-Value
**response to stress and abiotic stimulus**			
JZ535996↑	Dehydration-responsive element binding protein	*Krascheninnikovia arborescens*	3 × 10^−98^
JZ535839↑	Stress-induced protein sti1-like protein	*Atriplex canescens*	1 × 10^−161^
JZ536071↑	Manganese tolerance protein	*Beta vulgaris*	2 × 10^−112^
**cellular component organization and biogenesis**			
JZ536087↓	Non-specific lipid-transfer protein-like protein	*Vitis vinifera*	5 × 10^−37^
**transport**			
JZ535867↑	Bidirectional sugar transporter SWEET1-like	*Glycine max*	1 × 10^−110^
**response to endogenous stimulus**			
JZ535960↑	Ethylene response factor 3	*Malus x domestica*	3 × 10^−21^
**lipid metabolic process**			
JZ535825↑	Abscisic acid stress ripening protein	*Salicornia brachiata*	3 × 10^−20^
JZ535968↑	Glycine and proline-rich protein	*Ipomoea batatas*	0.62
**cell death**			
JZ535907↑	Leucine-rich repeat receptor-like protein kinase	*Theobroma cacao*	2 × 10^−105^
**thylakoid**			
JZ535969↓	Chlorophyll a/b binding protein	*Amaranthus hypochondriacus*	0.0
JZ535848↑	23 kDa Precursor protein of the oxygen-evolving complex	*Salicornia europaea*	4 × 10^−138^
**protein binding**			
JZ536063↑	General transcription factor IIE subunit 1-like	*Vitis vinifera*	7 × 10^−31^
JZ535986↓	Ankyrin domain protein	*Nicotiana tabacum*	2 × 10^−148^
JZ535815↑	Ubiquitin	*Medicago truncatula*	1 × 10^−161^
JZ536095↑	Dof-type zinc finger domain-containing protein	*Arabidopsis lyrata*	1 × 10^−30^
**catalytic activity (partly)**			
JZ536113↑	NADH dehydrogenase	*Brachypodium distachyon*	3 × 10^−64^
JZ536089↓	S-adenosylmethionine synthase	*Atriplex nummularia*	0.0
JZ536067↑	3-ketoacyl CoA thiolase	*Petunia x hybrida*	4 × 10^−120^
JZ535984↑	Short chain alcohol dehydrogenase-like	*Arabidopsis thaliana*	6 × 10^−65^
JZ536011↑	Chitinase	*Chenopodium amaranticolor*	2 × 10^−123^
**transporter activity**			
JZ535943↑	Aquaporin	*Knorringia sibirica*	1 × 10^−159^
JZ535964↓	Early nodulin 55-2 precursor	*Ricinus communis*	2 × 10^−33^
JZ535896↓	Sodium-bile acid cotransporter	*Ricinus communis*	5 × 10^−120^

↑, expression level of genes were up-regulated under salt; ↓, expression level of genes were down-regulated under salt.

### 2.5. Expression Level of Salt-Responsive Genes in A. canescens Using Quantitative RT-PCR

**Figure 6:**
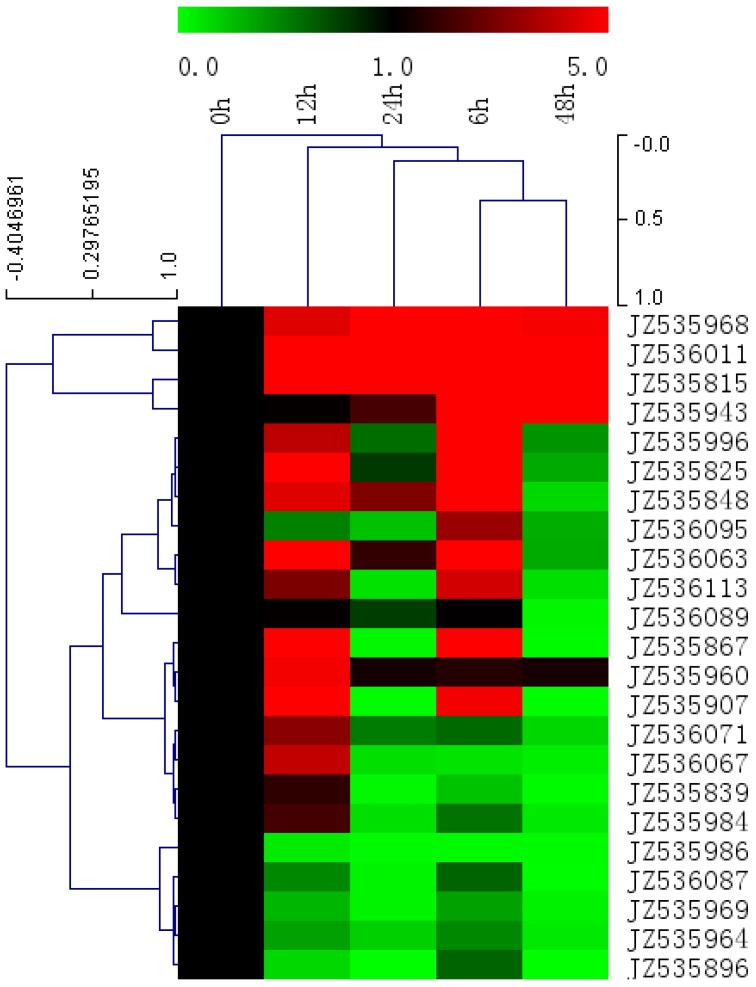
Hierarchical cluster of 23 potentially salt stress-responsive genes in transcript abundance with different times of 400 mM NaCl treatment (0, 6, 12, 24, and 48 h). Each gene is represented by a single row of colored boxes, and a single column represents different times with NaCl treatment. Induction (or repression) ranges from pale to saturated red (or green) with a fold change scale bar (in log2) shown up the clusters.

Among the ESTs that matched genes with known or putative functions, approximately 23 unigenes are involved in salt stress and defense according to the functional category and plant go slim. The results for gene expression show that 17 genes among the 23 genes were up-regulated and only 6 were down-regulated in response to NaCl treatment; these 17 genes may play important roles in abiotic stress in *A. canescens* (Figure 6, Table 1).

**Figure 7:**
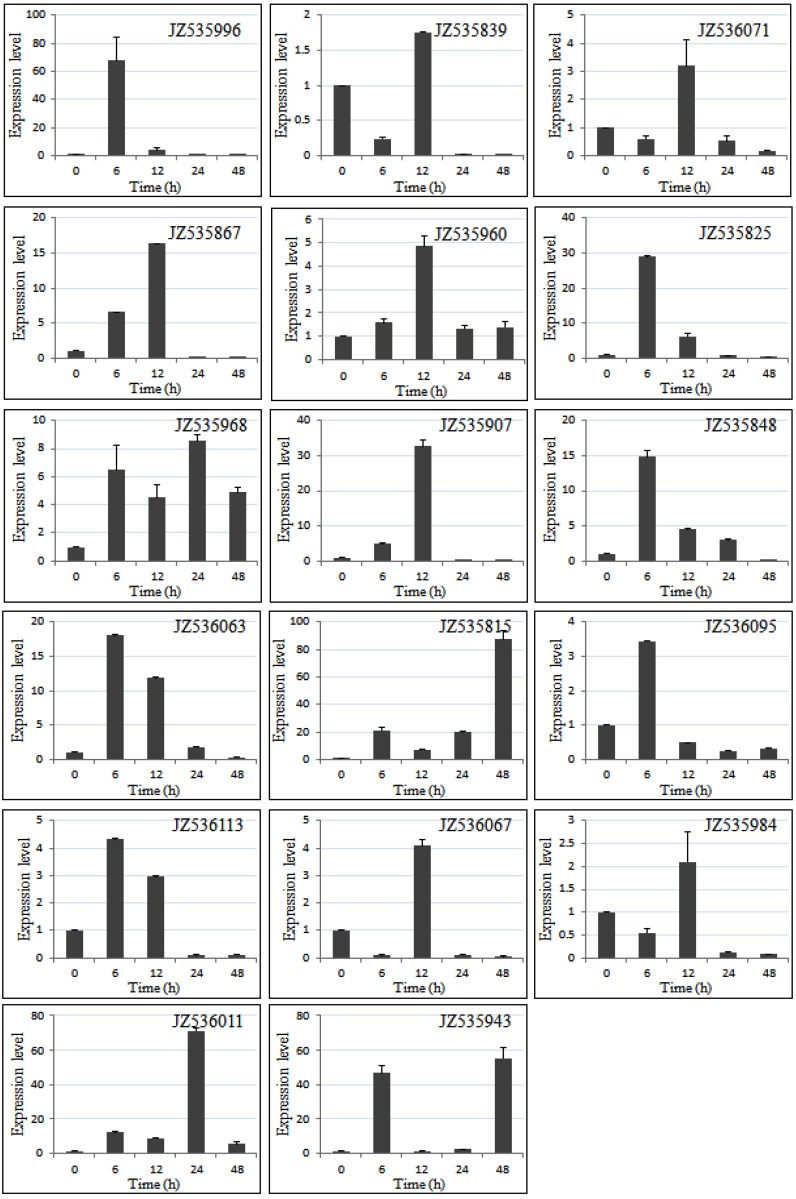
Quantitative RT-PCR validation of salt-related genes in the *A. canescens* with different times of 400 mM NaCl treatment. EF1α was used as internal control. The expression level means fold-changed obtained by quantitative RT-PCR.

There were 12 genes up-regulated at 6 h, and 13 genes at 12 h of salinity stress. However, the expression levels of only 5 genes were increased at 24 h and 4 genes at 48 h under the same conditions. Thus, most of the genes positively responsive to salt were upstream in the signal pathway such as: JZ535996 (dehydration-responsive element binding protein), JZ535960 (ethylene response factor 3), JZ535825 (abscisic acid stress ripening protein), JZ535907 (leucine-rich repeat receptor-like protein kinase), JZ535848 (23 kDa precursor protein of the oxygen-evolving complex), JZ536063 (general transcription factor IIE, subunit 1-like), JZ536095 (Dof-type zinc finger domain-containing protein), JZ536113 (NADH dehydrogenase), JZ536067 (3-ketoacyl CoA thiolase) and JZ535984 (short chain alcohol dehydrogenase-like) (Figure 7, Table 1). Among them, the relative expression level of three genes: JZ535996 (dehydration-responsive element binding protein), JZ535825 (abscisic acid stress ripening protein) and JZ535907 (leucine-rich repeat receptor-like protein kinase), was increased more than 30 times. These three genes may play important roles in the tolerance of plants during earlier stages of salt stress. The later stage responsive genes, such as JZ535815 (Ubiquitin), JZ536011 (chitinase) and JZ535943 (aquaporin), were significantly highly expressed, which may crucially contribute to the salt stress tolerance in *A. canescens.*

The first three identified early-responsive genes are “regulators” influencing down-stream genes. In contrast, three identified late-responsive genes are “structural genes”. These results support that the first early-reaction of plants was for osmotic shock while the late-reaction of plants was for strong salinity stress [7]. Further, it cannot be ignored that there are six down-regulated genes which may be also very important as results of strong salt stress/osmotic shock. For example, some Transcription factors (TF) can act as a “Blocker” or “Suppressor” regulating other structural genes. Therefore, if the expression of such TFs were down-regulated, the activation of the expression of down-stream genes, such as JZ535969 (chlorophyll a/b binding protein), JZ536089 (*S*-adenosylmethionine synthase) and JZ535964 (Early nodulin 55-2 precursor) can be observed. Interestingly, the ankyrin domain protein (JZ535986), as a nuclear transcription factor that negatively regulates the expression of cardiac genes, may play an important role in endothelial cell activation, and induction seems to be correlated with apoptotic cell death in hepatoma cells [30,31]. In contract, the sodium-bile acid cotransporter (JZ535896) and non-specific lipid-transfer protein-like protein (JZ536087) genes potentially playing an important role as ion antiporter were induced only weakly in the salt shock [7].

### 2.6. Identification and Characterization of SSRs

Table 2 shows the type and position of SSRs in the gene sequences. In total, 21 sequences containing 22 SSRs were identified from 343 consensus sequences, with one of the EST sequences containing two SSR. Analysis of these SSR motifs revealed that the proportion of SSR unit sizes was not evenly distributed. Most of these satellites are di- or tri- nucleotide motifs, being 15 (68.2%) and 6 (27.3%), respectively. There was only one occurrence of a tetranucleotide motif (4.5%). CT/AG was the most frequent repeat motif and accounted for 31.8% (7/22), followed by GA/TC (22.7%, 5/22), AT (13.6%, 3/22), TCA (9.1%, 2/22), and AAT (4.5%, 1/22), CAA (4.5%, 1/22), GAT (4.5%, 1/22), GTG (4.5%, 1/22). This is in agreement with a majority of studies that report dinucleotide repeats were the most abundant class of SSRs in sesame [21]. Most of the trinucleotide motifs were found only once. The mean SSR length of each unit varied between 10 and 27 bp. The overall average of SSR length was 19 bp with a maximum of 27 bp trinucleotide repeat (CAA).

The majority (50%, 11/22) of the identified SSRs are present in the within ORF, including all dinucleotide and trinucleotide repeats. Dinucleotide (TC/GA) repeats, are found in the coding regions and untranslated regions. SSRs are 6 in the 5'UTR and 5 in the 3'UTR. Additionally, among the 21 unigenes containing SSRs, 4 were unknown, others were stress-responsive genes, such as signal transduction and cell communication proteins (JZ535808, phosphate-induced protein; JZ535875, Jasmonate-induced protein), catalytic activity (JZ535928, arginine decarboxylase; JZ536029, JZ535877, DEAD-box ATP-dependent), Thylakoid (JZ535828, chlorophyll a/b binding protein; JZ535812, ATP synthase subunit), secondary metabolism proteins (JZ536018, thioredoxin H9-like), transferase proteins (JZ536097, 3-ketoacyl-CoA synthase; JZ535835, Serine hydroxymethyltransferase; JZ535901, endoplasmic reticulum-type calcium-transporting ATPase), Cellular component proteins (JZ536002, light-harvesting complex; JZ536047, nascent polypeptide-associated; JZ536117, RuBiSco large subunit-binding), kinase activity (3-hydroxy-3-methylglutaryl CoA reductase) and protein binding (JZ535947, polyubiquitin-like; JZ535851, metal ion binding protein). Interestingly, more than three-quarter of SSRs were present in the 5'UTR and within ORFs. Thus, most of these unigenes that contain SSRs are present in the 5'UTR or within ORFs, which indicated that these SSRs may be involved in regulating expression of these genes or enhancing protein functions [32].

**Table 2:** Frequency of EST-SSRs found in dbEST sequences and Distribution of SSRs with respect to putative open reading frames (ORF).

Sequence Accession No.	Repeat Motif	Repeat Numbers	Within ORF	5'UTR *	3'UTR *	Motif No. (Total, %)
JZ535808	CT	5	1			
JZ535828	CT	8			1	
JZ535877	CT	5	1			
JZ535901	CT	6	1			
JZ536002	CT	7			1	
JZ536029	CT	5	1			Di-
JZ536099	AG	5		1		(15, 68.2%)
JZ535947	TC	5		1		
JZ536047	TC	8		1		
JZ536117	TC	7		1		
JZ535983	GA	5	1			
JZ535992	GA	5			1	
JZ535812	AT	7			1	
JZ535835	AT	5	1			
JZ536041	AT	5		1		
JZ536078	TCA	6		1		
JZ536097	TCA	5	1			
JZ535851	AAT	8	1			Tri-
JZ536097	CAA	9	1			(6, 27.3%)
JZ536018	GAT	5	1			
JZ535928	GTG	6	1			
JZ535875	AAAC	5			1	Tetra-
						(1, 4.5%)
Total (%)	-	-	11 (50%)	6 (27.3%)	5 (22.7%)	22

* UTR, untranslated regions; Di-, Dinucleotide; Tri-, Trinucleotide; Tetra, Tetranucelotide.

## 3. Experimental

### 3.1. Plant Growth Conditions, Treatments and cDNA Library Construction

The *A. canescens* plants used for RNA preparation were grown in the greenhouse under controlled environmental conditions: 21 to 23 °C, 100 μmol·photons.m^−2^·s^−1^, 60% relative humidity, 14 h light/10 h dark in a Hoagland solution [33]. Plants were spotted with Peat and vermiculite (1:1), and watered with Hoagland solution prior to treatment twice per week. After 50 days of growth, plants were shifted to 2 L containers with Hoagland solution and aerated hydroponics, and the pH was 6.0. After 3 days for adapting the hydroponic conditions, plants were transferred into fresh solution with 400 mM NaCl in one step [7]. After 48 h treatment, the harvested samples (young leaves, stems, fibrous roots) were immediately frozen with liquid nitrogen and kept at −80 °C, for use for the RNA extraction and cDNA library construction. Total RNA was extracted from *A. canescens* samples mixture using a Trizol reagent kit (Invitrogen, Carlsbad, CA, USA). RNA quality was checked by spectrophotometry, while its integrity was verified on agarose gel. The mRNA for the cDNA library construction was isolated using a FastTrack^®^ 2.0 Kit (Invitrogen) according to the manufacturer’s instructions. The uncut cDNA library was synthesized with a Superscript Full length library construction kit II (Invitrogen) following the manufacturer instructions, and ligated into a pDONR222 entry vector. Then the cDNA inserts were introduced into the vector pYES-DEST52 (Invitrogen) via LR reaction and subsequently transformed into *E.coli* DH5α cells.

### 3.2. cDNA Sequencing Strategy

The *E.* *coli* DH5a cells carrying cDNA library were plated onto Luria-Bertani (LB) agar plates (ampicillin, 100 mg/mL). Colonies were picked randomly and transferred to a 1.5 mL Eppendorf tube containing 0.6 mL LB media supplemented with ampicillin, and incubated in a horizontal shaker at 200 rpm and 37 °C overnight. The size of the insert fragment and the recombinant rate were measured by PCR. PCR amplification was carried out on a BIO-RAD 5100 Thermal Cycler in 25 μL reaction mixtures (18.0 μL ddH_2_O, 2.5 μL 10× Tag PCR buffer, 1 μL 10 mM dNTP mixture, 1 μL 10 μM each PCR primer, 0.5 μL Taq DNA polymerase (5 U/μL), and 2 μL of overnight suspension from a single bacterial colony as template), which followed the reaction procedure: 5 min at 94 °C for initial denaturation; 30 cycles of 30 s at 94 °C for denaturation, 45 s at 58 °C for annealing, and 4 min at 72 °C for extension; 10 min at 72 °C for final extension and then kept at 4 °C. Sequencing reactions were performed using T7 primer (5'-TAATACGACTCACTATAGGG-3') according to pYES-DEST52 vector. 

### 3.3. Sequence Processing and Analyses

Poor-quality sequences, or sequences with less than 100 bases, and vector sequences were trimmed from the raw single-pass sequences using SeqMan II (DNASTAR, Inc., Madison, WI, USA) and NCBI VecScreen [34]. All consequential EST sequences were deposited in the GenBank dbEST database and were subjected to data analyses.

The trimmed cDNA sequences were assembled into clusters using the assembly program within SeqMan II set to default parameters. Contigs were built using the CAP3 assembly program [35] with the parameters set at 95% identity over 40 bp. Individual tentatively unique genes were subjected to BLASTX analysis against the non-redundant (nr) database [36]. Unigenes (contigs and singletons) were annotated using BLASTX against the NCBI non-redundant protein database with a cut-off E-value of the best hit of ≤10^−5^ [24]. Sequences without a reliable match (>10^−5^) were subsequently compared with the NCBI non-redundant nucleotide database by performing BLASTX (score > 100) for complementary annotation [37]. 

### 3.4. Functional Annotation and Functional Categorization

Identified sequences were divided into 8 functional categories based on the Arabidopsis MIPS functional category denomination [38]. All well-annotated unigenes were then further classified and mapped to the three Gene Ontology (GO) categories (biological, cellular, and molecular) via AmiGO [39]. The GO terms ID, CateGOrizer [40] was used to classify GO terms in plant GO slim classes and give a broad overview of the ontology content without the specific fine-grained terms. The ESTs were further analyzed to identify putative abiotic stress-related genes.

### 3.5. Quantitative RT-PCR Validation of Salt-Related Genes

*A. canescens* total RNA with different times of 400 mM NaCl treatment (0, 6, 12, 24 and 48 h) were harvested as described in Experimental Section 3.1. For real-time quantitative PCR, 2 µg of total RNA was used to generate cDNA with a SuperScript First-Strand Synthesis System kit (Invitrogen). Quantitative expression assays were performed with the SYBR^®^Green Reagent kit on the 7500 real-time PCR detection system according to the manufacturer’s protocol (Applied Biosystem, Foster City, CA, USA). Each reaction was done in triplicates with a reaction volume of 20 µL. qRT-PCR conditions were as follows: 30 s at 95 °C; 40 cycles of 5 s at 95 °C, 34 s at 60 °C; 15 s at 95 °C, 60 s at 60 °C, 15 s at 95 °C; 15 s at 60 °C. Samples were run in technical replicates on each 96-well plate. The relative quantification method (2^−ΔΔ*C*t^) was used to evaluate quantitative variation between replicates [41], and the Elongation Factor 1-alpha (EF1α) gene was used as a house-keeping gene to normalize all data. The primer pairs used for real-time PCR are listed in Supplementary Table S3. Hierarchical clustering was performed using the program [42]. Clustering was based on the quantitative RT-PCR measure, experiments were carried out in triplicate and representative clusters are shown.

### 3.6. Frequency and Distribution of EST-SSRs Found in dbEST Sequences

Potential SSRs (simple sequence repeats) markers were detected among the 343 unigenes using the tool of SSRIT (Simple Sequence Repeat Identification Tool) online [43] with a repeat motif length of two to six nucleotides sequences. Mononucleotide repeats were ignored since distinguishing genuine mononucleotide repeats from polyadenylation products and single nucleotide stretch errors generated by sequencing was difficult. The minimum repeat unit was defined as five for dinucleotides, tri-nucleotides and four for tetra-, penta-, and hexa-nucleotides [21].

To predict the position of SSRs with respect to coding regions, the open reading frames (ORFs) were identified. ORF prediction was based on ORFfinder tools [44] and BLASTX hits. The part of the ORF that matched the best BLASTX hit was considered as seed to select the right coding region from the six frame translations provided by ORF finder. Based on the both results, we defined the beginning and the end of the ORF [32]. Usually an open reading frame starts with an ATG (methionine) and ends with a stop codon (TAA, TAG or TGA).

## 4. Conclusions

*Atriplex* species are well adapted to both salt and low-temperature stresses and can serve as one of the model species to understand mechanisms of tolerance in plants [45]. Very little research has been carried out to identify the molecular mechanisms directly responsible for the specific tolerance of *Atriplex* species to abiotic stress [2]. We present here the analysis of a high quality cDNA library and the ESTs from *A*. *canescens* grown under salt conditions. The primary titer of the cDNA library was 1.76 × 10^6^ CFU, with a recombinant rate of 91%. The sizes of the inserts ranged from 0.4 to 2.4 kb, and the average insert size was estimated to be 1.25 kb. The aim of this study was to generate a large amount of high-quality ESTs that would constitute a good basis for future more detailed studies in *A*. *canescens*, and to give an initial view of gene expression and identify novel abiotic tolerance genes in *A*. *canescens* under salt stress. In an evaluation of 343 valid EST sequences in the *A*. *canescens* cDNA library, 197 unigenes were assembled, among which 190 unigenes (83% ESTs) were identified according to their significant similarities with proteins of known functions. Ten most abundant genes in the EST collection accounted for 32.28% of the total ESTs. All the 343 EST sequences have been deposited in GenBank under accession numbers JZ535802 to JZ536144.

According to *Arabidopsis* MIPS functional category GO classifications, we identified 193 unigenes of the 311 annotations EST, representing 72 non-redundant unigenes, that share similarities with genes related to defense and stress response, and some novel ESTs were also obtained. Further investigations in gene functional characterization would help to discover promising candidates with a key role in development under stresses, and most of the true physiological role of genes potentially involved in the tolerance to abiotic stresses has yet to be determined.

Due to the weakness of the GO databases, there is low reliability for their use to determine that these genes are responsive to salt stress. Therefore, further research such as expression analysis of these selected genes in salt stress condition using quantitative real-time PCR were performed. The results show that 17 genes were positively regulated and 6 genes were negatively regulated. Six of the identified 17 genes may play important roles in the tolerance to salt stress in *A. canescens*, among them 3 were in earlier stage responsive genes and 3 others were later stage responsive genes. Expression profiles of other genes indicated their increase but not significantly. However, such genes may also help to improve salt tolerance in some other pathways.

SSRs derived from ESTs essentially represent expressed genetic sequences and hence are potential candidates for the construction of markers for gene tagging and comparative genomic studies. However, the exact function and occurrence of these genes expressed in response to salinity and contained SSR fragments need to be further characterized. The identification and the study of these stress-responsive genes and gene-based functional markers may provide a shortcut to investigate the mechanism of the requirement to salinity stress tolerance in *A. canescens.*

Thus, ESTs data obtained here from *A*. *canescens* should be a useful tool for abiotic stress tolerance research on halophytes. The discovery of novel genes controlling tolerance to abiotic stresses is important for cultivating transgenic plants with potential tolerance to multiple abiotic stresses.

## Supplementary Files

Supplementary File 1 Supplementary Information (PDF, 702 KB)
