# Supplementary material for: Generation and Analysis of Expressed Sequence Tags (ESTs) from Halophyte Atriplex canescens to Explore Salt-Responsive Related Genes
Source: Int J Mol Sci. 2014 Jun 23;15(6):11172–89. doi: 10.3390/ijms150611172 (PMC4100206; doi:10.3390/ijms150611172)
Supplement: Supplementary File 1 — Supplementary Information (PDF, 702 KB) [file ijms-15-11172-s001.pdf]

Supplementary Information

Figure S1. Agarose gel electrophoresis analysis. (**A**) Total RNA extracted from materials treated with NaCl stress 48 h; (**B**) PCR analyses of partial cDNA insert fragments, lanes 1 to 32 = single colony; (**C**) MW, 1 kb Plus DNA ladder (Invitrogen).

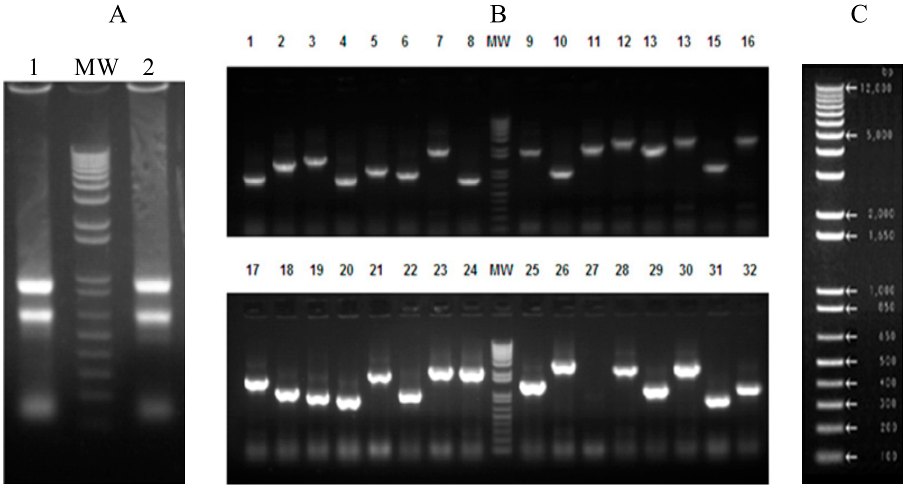

Table S1. General characteristics of *A. canescens* ESTs.

Items	Number
Total sequenced cDNA	413
Total number of ESTs analyzed	343
Total reading valid length (bp)	322,524
Average EST length (bp)	940
G + C content (%)	44.66
Unigenes	197
Contigs	50
Singletons	147
Redundancy [a]	42.6%

Redundancy [a] = number of Unigenes assembled in total ESTs; The redundancy of the library was calculated as [(1 − Number of Unigenes/Number of ESTs) × 100%].

Table S2. Abundant ESTs found in *A. canescens* DNA library.

Putative Function (E-Value < 10^{-4})	Number of ESTs	Percentage of Total *
Uncharacterized classification	58	16.9
Chlorophyll A/B binding protein	10	2.62
Heat shock	8	2.33
Glyceraldehyde-3-phosphate dehydrogenase	8	2.33
Ribosomal protein	6	1.74
Ubiquitin	5	1.45
Eukaryotic elongation factor	5	1.45
Early light induced protein	4	1.16
DEAD-box ATP-dependent RNA helicase	4	1.16
Oxygen-evolving enhancer protein	4	1.16
Total	108	32.28

* Percentage = Number of ESTs/Number of total ESTs × 100%

Table S3. Pair of primers of salt resistance-related genes and reference gene for quantitative RT-PCR (in the order of accessions presented in Table 1).

Accession NO.	Genes Description	Forward Primers	Reverse Primers
JZ535996	Dehydration-responsive element binding protein	5'-CTCTAACCCGATGCCTGCTT-3'	5'-CTCTTCCCGCTGTTCTCCTG-3'
JZ535839	Stress-induced protein sti1-like protein	5'-TAAAGCAAGCCGTGGGGATT-3'	5'-GCTGCCTTAGGGTTCTCCTG-3'
JZ536071	Manganese tolerance protein	5'-TGGAACCACCACAAGGCAAT-3'	5'-TCGAGGTGAACAAAAGCACG-3'
JZ536087	Non-specific lipid-transfer protein-like protein	5'-AGCAAGCTAGGCGATGTTGT-3'	5'-TGGACCTTGCAAGCAGTAGG-3'
JZ535867	Bidirectional sugar transporter SWEET1-like	5'-TCTTCTTGCTCCCCGAAACT-3'	5'-GCATTAGCAGGCTTGATCGC-3'
JZ535960	Ethylene response factor 3	5'-CGAGCTCGATGGCTCAAGCTATGGC-3'	5'-CGGGGTACCACCAAGCTTGGCAAGAACA-3'
JZ535825	Abscisic acid stress ripening protein	5'-ATACACCGAGACCACCACCA-3'	5'-AGCTCACCCAAATGCTCCTT-3'
JZ535968	Glycine and proline-rich protein	5'-GCTGGTTATCCAGGGGGTTC-3'	5'-TTCCCATGCTTCCCATGCTT-3'
JZ535907	Leucine-rich repeat receptor-like protein kinase	5'-GCCGTCAAGGTGCTCTCTAA-3'	5'-GCTGGGTCCTTCTTCACAGT-3'
JZ535969	Chlorophyll a/b binding protein	5'-CGGTGGTAGCTTTGACCCAT-3'	5'-GGCCCTTTCCGGTGACAATA-3'
JZ535848	23 kDa Precursor protein of the oxygen-evolving complex	5'-GGTTGGCTCTCACTGTCCTC-3'	5'-AATCCTTCGCCGTTGTAGGG-3'
JZ536063	General transcription factor IIE subunit 1-like	5'-AAGGGCATGATTCCGATGCT-3'	5'-GGCTGCAGGCCTTGATGATA-3'
JZ535986	Ankyrin domain protein	5'-GTTCTGTGCCAGACGAGTCA-3'	5'-CCCTCCACATCACCAATGCT-3'
JZ535815	Ubiquitin	5'-AGAGACTATGACCAGGCGGT-3'	5'-GTCCTCCCATCCTCCAGACT-3'
JZ536095	Dof-type zinc finger domain-containing protein	5'-GATGGATGCTGTGCTCCCTT-3'	5'-AGTGTTACCATGCCCATCCG-3'
JZ536113	NADH dehydrogenase	5'-TAAGCCTTGCCTTCCTCAGC-3'	5'-CGTGGGTTACCCTTTACCCG-3'
JZ536089	*S*-adenosylmethionine synthase	5'-ATGGTACCTGCCCATGGTTG-3'	5'-TCCTTCAAGTCAGCGGCAAT-3'
JZ536067	3-Ketoacyl CoA thiolase	5'-GATGAAGCGCCGTGGTAAAG-3'	5'-ACGAGCGTTGCTTAGGTCAT-3'
JZ535984	Short chain alcohol dehydrogenase-like	5'-ATTTGGCATGTGAATGGGCG-3'	5'-CGAGACTCGACTGCCTTCAA-3'
JZ536011	Chitinase	5'-AAGTTCGGGACTGGTGGTTC-3'	5'-CCCAATGCTTTTACCCGCTG-3'
JZ535943	Aquaporin	5'-CCTCTACATAACCGTCGCTAC-3'	5'-TTCACCAAACCCACTCCA-3'
JZ535964	Early nodulin 55-2 precursor	5'-AATCTTCGCAGCAACAGC-3'	5'-CCAACACCAACTGAAGGAA-3'
JZ535896	Sodium-bile acid cotransporter	5'-GGAAATGTTGCGCTCTCAGT-3'	5'-TGGGCTTGGGCTAAATGAGT-3'
KJ027085	EF1a	5'-CCCCAGTTCTCGACTGTCAC-3'	5'-TGGTGGGAACCATCTTCACG-3'
